# Effect of Music on Stress Parameters in Dogs during a Mock Veterinary Visit

**DOI:** 10.3390/ani12020187

**Published:** 2022-01-13

**Authors:** Tammie King, Hannah E. Flint, Alysia B. G. Hunt, Walter T. Werzowa, Darren W. Logan

**Affiliations:** 1 Waltham Petcare Science Institute, Freeby Lane, Waltham on the Wolds, Leicestershire LE14 4RT, UK; hannah.flint@effem.com (H.E.F.); alysia.hunt@effem.com (A.B.G.H.); darren.logan@effem.com (D.W.L.); 2 HealthTunes Inc., 1800 S. Brand Boulevard Suite 114, Glendale, CA 91204, USA; walter@healthtunes.org

**Keywords:** dog, stress, anxiety, veterinary, behavior, fear, music

## Abstract

**Simple Summary:**

Visits to the vet are stressful for many pet dogs, but less is known about how measures of stress change over the course of a visit. Identifying appropriate measures of canine stress, along with successful interventions which alleviate stress in dogs during a veterinary visit, will be of great benefit to dogs and people. Music therapy has been successfully used to reduce stress and anxiety in people and other animals. Specifically, a process called entrainment, which involves playing music at a particular tempo aimed at synchronizing physiological responses, has been implemented with success in humans. The aim of this study was to examine a range of behavioral and physiological measures in dogs over the duration of a veterinary visit and to establish if bespoke music, which mimicked the tempo of their resting heart rate, could improve wellbeing. The results indicated that certain measures increased over time, indicating that dogs became increasingly stressed. Music was not shown to have a demonstrated effect across measures, suggesting that the stressor may be too extreme for this type of intervention to have a positive effect, or that music therapy requires modification before it can be successful in alleviating stress in dogs during a veterinary visit.

**Abstract:**

Veterinary visits can be stressful for dogs, but how their wellbeing changes during a visit is not well understood. Music therapy has been successfully used in clinical practice to alleviate stress and anxiety in people. The present study aimed to understand how canine stress changes during a veterinary visit, establish the effect of music, and highlight measures which may be of practical use. In a randomized crossover design, dogs were exposed to no music and a bespoke piece of classical music at a tempo designed to match their resting heart rate during a mock veterinary visit. Dogs were scored as more “afraid” during the physical examination compared to when they were in the hospital kennel (*p* < 0.001). Salivary cortisol, IgA, and infrared temperature all increased significantly (*p* < 0.05) from baseline to post-kennel and post-examination, with no effect of music treatment. Core body temperature (*p* = 0.010) and the odds of ‘relaxed’ lips (*p* = 0.020) were lower when dogs were exposed to music compared to control visits. Overall, dogs experienced changes in physiology and behavior, indicative of increased stress, over the course of the visit. Additional research is required to further understand the effect that bespoke music may have in alleviating canine stress during veterinary visits.

## 1. Introduction

Veterinary hospitals are considered stressful environments for dogs [1,2,3,4,5,6] as well as caregivers [7], and these negative experiences can have long-lasting effects on animals [8]. Although the mammalian stress response is an adaptive mechanism that allows animals to respond quickly to certain situations or stimuli, chronic or extreme stress, often categorized as distress, can be harmful to animals, resulting in poor welfare [9]. Although it is widely accepted by owners and practitioners that dogs experience stress and anxiety when visiting a veterinary clinic, to what extent a pet may be impacted is not fully understood. Possible reasons for this may be because there are no clear protocols specifying how canine stress can be accurately measured and monitored in clinical environments, along with the accompanying complexity of capturing data across different timepoints during a visit, and the inability to obtain output in a timely manner. Commonly, data are collected in “real-world” environments where standardization may be lacking and added variables, such as the presence of other pets, staff, and the owner, can influence measures. The experience and handling skills of both veterinary staff and pet owners may also influence pet behavior and physiology, along with the different types of treatment protocols and whether the dog is in pain. Traditional measures include owner or staff questionnaires [10], ratings of stress [5], and coding of specific behaviors, e.g., lip licking, whale eye, body posture, panting, and trembling [11], as well as a range of physiological parameters. These can include eye temperature using infrared thermography (IRT) [4], heart rate, heart rate variability, blood pressure, along with salivary or plasma cortisol and immune markers such as immunoglobulin A (IgA), as well as neutrophil–lymphocyte (N:L) ratios. (For a detailed overview of physiological measures of stress in dogs, see Hekman et al. [12]). Some parameters are difficult, costly and/or time consuming to capture and can be influenced by circadian rhythms, physical health, and activity [12]. For example, the collection of behavioral data via video recordings is relatively straightforward but resource intensive when objectively coding specific behaviors. Conversely, owner-directed questionnaires may be a relatively quick and easy means to obtain data; however, they are subjective in nature and therefore potentially inaccurate [11]. An increasingly popular approach is to use instruments that require ratings of specific behaviors or behavioral attributes that can be scored by an experimenter [13]. Qualitative Behavior Analysis (QBA) [14] yields accurate and repeatable results to infer animals’ affective states. Unlike specific coding of individual behaviors, QBAs act as a “whole-animal” measure which captures a holistic view of an individual’s demeanor [15]. However, to best understand an animal’s emotional state, the ideal approach is to combine easy-to-administer scales which evaluate behavior, along with a range of physiological parameters.

It would be beneficial to identify interventions that are easy to implement and supported by science that can improve pet wellbeing in a veterinary setting. This has the potential to be multi-beneficial. Caregivers are likely to feel more at ease and may therefore be more likely to return to a clinic that prioritizes their pet’s welfare. Animals who are less stressed, anxious, or afraid are less likely to exhibit aggressive behavior [16] which may result in staff injuries.

A range of strategies to make vet visits a more pleasant experience for pets are recommended by practitioners who take an interest in animal welfare, including low-stress handling techniques, appropriate waiting/consultation/hospital room layout, pheromonotherapy, lighting, and aromatherapy [8,17,18]. An approach which has not been widely recommended, employed, or empirically tested is the inclusion of music therapy in veterinary hospitals [19]. Often, veterinary clinics have a radio or television playing in their waiting rooms, and music may be played during surgical procedures, but this is generally for the benefit of staff or clients, rather than specifically to aid in pet welfare. Music has been used for centuries to influence human health [20], including as a form of alternative therapy to reduce patient pain [21], stress, and anxiety [22] and to improve staff and patient wellbeing [23]. The exact mechanisms of these therapies are not well understood, but one possible explanation is that music acts as a distractor stimulus, focusing the patient’s attention away from fear- or pain-inducing stimuli to something pleasant and enjoyable. Much less is known as to whether music has similar effects on non-human animals, such as cats and dogs, in clinical environments. Classical music, in particular, is thought to have positive effects in a veterinary setting, but empirical studies to support this tend to be weak [24]. Assumptions are often based on other studies which include healthy animals not representative of hospitalized patients or those within a clinical environment, sample sizes are often small, with lack of randomization and inconsistent treatment approaches. However, one study demonstrated that cats who are exposed to classical music whilst under anesthesia showed reduced respiratory rate and pupil diameter when undergoing painful surgical procedures [25]. Another study demonstrated that classical music improved owner satisfaction during a veterinary visit [26]. Other studies have focused on the effect music has on laboratory animals or livestock, concluding that some forms of music improve milk production and meat quality, while also decreasing potential indicators of stress, such as heart rate and blood pressure [27]. More commonly, research involving music and companion animals has focused on pet welfare in kenneled environments (working and shelter dog populations) [28,29,30,31,32,33]. Results from these studies have indicated that the type of auditory stimuli is important, with kenneled dogs showing more calm behaviors when exposed to classical music [33], and more active behaviors when exposed to heavy metal music [29]. A separate study found that kenneled dogs displayed more relaxed behavior when exposed to audiobooks than when exposed to any other auditory stimuli [30]. It may not necessarily be the classification of music that is important, but the structure of the piece of music related to its notes, tone, rhythm, and tempo [34], along with the individual dog’s previous associations with the music. A study which examined the effect certain music types had on felines showed that cats prefer species-specific music, so sounds that were at a higher pitch and tempo [35]. When tested in a clinical setting, cats were scored as being significantly less stressed and easier to handle compared to when they were exposed to classical music or no music [36]. In humans, music induces both arousal and relaxation effects, predominantly related to the tempo [37]. Slow or meditative music can induce a relaxing effect; relaxation is particularly evident during a musical pause, whereas faster rhythms concentrate attention [38]. Specifically, physiological measures including heart rate, blood pressure, and respiration modulate in response to music containing particular tempi. Another mechanism related to music tempo which has been demonstrated to positively affect humans is the process of entrainment. Entrainment describes a process whereby two or more rhythmic properties synchronize with one another [39]. Studies have demonstrated positive entrainment and stimulation caused by certain tempi and/or rhythms in chronic pain, sleep, anxiety, mobility, and heart rate treatments [40,41]. Rhythmic entrainment using music may also be beneficial in alleviating anxiety levels in dogs, attempting to synchronize biorhythms (such as heart rate) to bespoke music set to resting heart rate tempo.

To our knowledge, there have been no studies which examine the phenomena of music entrainment in dogs and its effect on reducing stress during a veterinary visit. To examine the topics of canine stress and music therapy in more detail, the present study was designed to address three key objectives. Firstly, to understand how specific measures of canine stress change during a mock veterinary visit, secondly, to establish the effect bespoke music has on stress parameters in a clinical environment, and thirdly, to provide recommendations on measures of canine stress for in-clinic use.

## 2. Materials and Methods

### 2.1. Subjects

Thirty-eight adult dogs, seventeen males and females of various breeds (14 Labrador Retrievers, 6 Beagles, 6 Petit Basset Griffon Vendéen, and 12 Norfolk Terriers), with a mean age (±sd) of 3.4 ± 1.9 years, ranging from 1 to 8.2 years, participated in the study. All dogs were housed in pairs within kennels at the Waltham Petcare Science Institute (Leicestershire, UK). Throughout the duration of the study, all dogs were provided with comprehensive training and socialization programs, adjusted to the needs of individual dogs as per the Institute’s standard pet keeping requirements. Prior to the study, dogs had been exposed to the testing environment and underwent basic mouth handling training to facilitate saliva sample collection.

### 2.2. Veterinary Clinic

The study was conducted in the veterinary suite located at the Waltham Petcare Science Institute between November and December 2019. The temperature-controlled veterinary clinic was a standard set-up including an office/foyer area, walk-in hospital kennels, consultation room, and surgery, along with diagnostic facilities and associated veterinary equipment. The experimental procedure was designed to simulate a veterinary clinic experience which comprised a period of “waiting” time in the hospital kennel area (1.59 m × 0.98 m), followed by a routine veterinary health examination in a consultation room (6.46 m × 2.94 m). The walk-in kennel had solid opaque walls with a transparent Perspex front door. Kennels faced into a hallway. No other animals or staff, other than those involved in the collection of data, were present in the veterinary clinic while the study was underway.

### 2.3. Study Design

Using a randomized crossover design, each dog experienced two mock veterinary visits which occurred at least two weeks apart, one where music was played and one without music. Bespoke pieces of classical music, composed by an experienced, professional music producer, were designed to match the tempo of the estimated resting heartbeat of each dog breed. In consultation with the site’s veterinarian, the heart rates of specific beats per minute (bpm) for small (120 (±20)), medium (100 (±20)), and large (95 (±20)) breeds included in the study were collected and used by the HealthTunes music producer when designing the music. Three curated playlists were provided for each of the dog sizes (Audio S1; Audio S2; Audio S3). Each playlist contained solo-harp music arrangements of classical works. The dynamic range of the music was narrow (no sudden volume peaks/accents), the arrangements fluid (no sudden pattern changes), and the frequency band well balanced (mid-range frequency dominates and high/low frequency bands evenly audible). The music program was mastered at −0.1 dB. During the experimental procedure, music was delivered continuously through two SONOS One wireless audio speakers mounted on the walls within the testing environment during treatment sessions. One speaker was placed in the kennel room hallway and the other inside the consultation room and provided stereo sound with the same music quality in both locations.

The standardized mock veterinary visit was separated into two phases—waiting time in the kennel area followed by a physical examination in a consultation room.

Dogs entered the foyer of the building with a familiar handler at the same time each day for their test sessions. Immediately after entering, a series of baseline measures were collected by a researcher (T0). The dog was then led by the handler into the walk-in kennel, where they were left alone for a duration of 15 min and exposed to music or no music (Kennel). Additional data were collected immediately after the end of this session (T1), and the dog was then led by the handler into the consultation room, where a second researcher, acting as a mock veterinarian, performed a routine health examination (Consult). To ensure consistency, the same researcher was used throughout. The handler and “veterinarian” engaged in small talk to mimic what would occur during a routine clinic appointment. Small and medium dogs (<15 kg) were picked up by the handler and placed on the examination table to receive their health check, while large dogs (>15 kg) received their health examination on the floor. During the examination, which lasted approximately 10 min, the mock veterinarian, who wore a white laboratory coat, followed a standardized procedure and accompanying script where they spoke to the dog’s handler whilst completing a health exam. They checked the dog’s eyes, mouth, nose, and ears, palpated lymph nodes and the abdomen, then provided a foreleg and foot exam. They also took each dog’s heart rate, respiration rate, and finally, rectal temperature. If at any point the dog displayed signs of distress, the examination was modified to maintain dog welfare and ensure each dog could participate as much as possible. Distress was considered as the prolonged duration, intensity, or frequency of any stress-related behavior such as trembling, panting, cowering, vocalizing, low body posture, and avoidance. Directly after the examination, additional data were collected (T2) before the dog was led back to the foyer and collected by a member of staff, where they were then returned to their familiar housing.

The study was reviewed and approved by the Waltham Petcare Science Institute Animal Welfare and Ethical Review Board (study number PPM 63406).

### 2.4. Data Collection and Analysis

**Figure 1:**
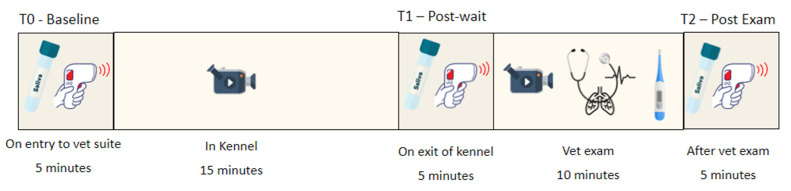
A range of physiological and behavioral data were collected at various timepoints during testing sessions.

A range of data were collected at three sample points: T0—baseline (after entering the foyer/office), T1—after being left alone in the kennel, and T2—after the physical examination in the consultation room. Behavioral measures were collected later from video recordings of the dog in the kennel (Kennel) and during the health examination in the consultation room (Consult) (Figure 1).

#### 2.4.1. Eye, Nose, and Ear Temperature

**Figure 2:**
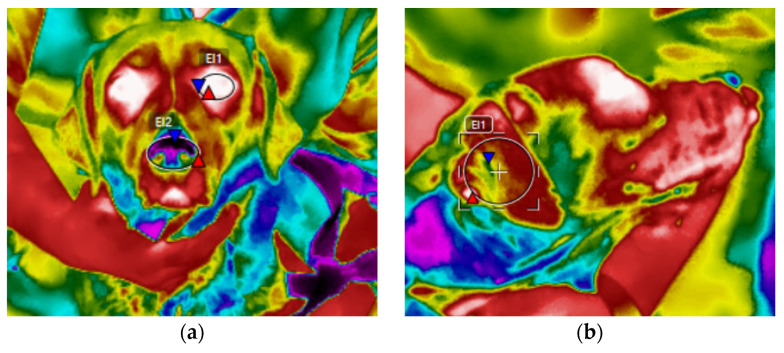
Infrared thermal images of a Labrador Retriever with ellipses drawn for (**a**) the eye and nose and (**b**) the ear flap.

Infrared thermography (IRT) was used to capture the mean eye and nose temperature and maximum and mean ear temperature at T0, T1, and T2. A portable infrared thermal camera (FLIR T840, FLIR, Wilsonville, OR, USA) was used to capture all infrared images during the study with a thermal range of—20 to 150 °C and a resolution of 464 × 348 pixels. The value of emissivity was set at 1. All dogs were pictured with the camera at a 90° angle to the dog and from approximately 1 m away. Once collected, images were analyzed using FLIR Tools software (FLIR, Wilsonville, OR, USA). Using the software, the following temperatures were taken: the mean temperature of an ellipse drawn within the anterior surface region of the eye; the maximum and mean of an ellipse drawn within the surface of the ear flap; along with the mean of an ellipse drawn encompassing the anterior surface of the nose (Figure 2).

#### 2.4.2. Salivary Cortisol and IgA

Saliva was collected at T0, T1, and T2 using SalivaBio Children’s Swabs (Salimetrics, Carlsbad, CA, USA) to measure cortisol and sIgA. No stimulus was provided (e.g., toys/food) to avoid impacting the measures collected. The swab was inserted briefly into the left-side buccal cavity area of the dog’s mouth for 30–60 s before the tip was cut off and placed into the collection tube. This was repeated with the other end of the swab for the right-side buccal cavity and below the tongue. Both tips were placed in the same tube. The collection tube was immediately placed on ice and transported to the onsite laboratory. Samples were centrifuged at 4 °C before being stored at −20 °C in preparation for later analyses. The expanded range high sensitivity salivary cortisol enzyme immunoassay kit by Salimetrics (Salimetrics, Carlsbad, CA, USA) was used as per the kit protocol with an intra-assay variation of 5.1% and a limit of quantification of >0.04 µL/dL. The sIgA was analyzed using the Abcam IgA Dog ELISA Kit with an intra-assay variation between 4.2 and 7.4% and a limit of quantification of 4.02 ng/mL. 

#### 2.4.3. Temperature, Pulse, and Respiration (TPR)

TPR measurements were collected by the mock veterinarian at the end of the physical examination within the consultation room. Rectal temperature was collected using a Genia Digiflash Thermometer, while pulse and respiration were collected using a stethoscope. Readings were documented on paper for each dog and later manually entered onto an electronic spreadsheet.

Dogs also wore PetPace^TM^ (Burlington, MA, USA) monitors to automatically capture heart rate and vasovagal tonus index (VVTI), a measure of heart rate variability, at 2 min intervals throughout the entire vet visit. A PetPace^TM^ monitor collar was placed on the dog whilst they were situated in their familiar housing, prior to being led by their handler to the veterinary suite. The collar was then removed immediately prior to leaving the veterinary suite upon the completion of testing.

#### 2.4.4. Dog Behavior

Dog behavior was recorded during the kennel wait time (Kennel) and the physical examination (Consult) using GoPro Hero 7 cameras mounted on tripods. The cameras were positioned in a standardized position in front of the kennel and either on the floor or on the examination table in the consultation room to best capture each individual dog for the duration of the experimental procedure.

Behavioral data were coded from video footage from the kennel room and consultation room. A QBA previously developed to evaluate dog welfare in shelter dogs [15] was modified slightly to include an additional term, “calm”, and was used to collect behavioral data during the dogs’ time in the kennel and during the health examination. The final QBA consisted of a list of 21 terms with associated characteristics that scorers used to rate each dog (Table 1). 

The QBA was used to assess the overall level of intensity at which certain attributes were present during the dog’s time alone in the kennel and throughout the physical examination. Three trained raters who were familiar with dog behavior and body language were randomly assigned videos so that the behavior of all dogs was scored. Each rater was provided with scoring sheets (one for each video clip) on which Visual Analogue Scales (VAS) with lengths of 125 mm were placed next to each term. Raters were instructed to independently score each dog on every qualitative term on the list. The left end of the VAS scale corresponded to the minimum score (0 mm), meaning the expressive quality indicated by the term was entirely absent in the dog, whereas the right end represented the maximum score (125 mm), meaning that the quality indicated by the term was strongly dominant in that dog. Raters watched each assigned video using their laptops, and after each clip, they were encouraged to score the dogs’ expressions on the rating scales as quickly as possible by marking a cross on the VAS at the point they felt was appropriate. A score was assigned to each term for each video by using a ruler to measure the distance in millimeters between the minimum point of the VAS and the point where the observer marked the line.

To capture additional behavioral data during the physical examination, a scale previously developed to specifically evaluate dog behavior during a veterinary consultation was used. The Clinic Dog Stress Scale (CDSS) [13] (Table 2) originated as a way to measure a dog’s behavior during an examination and is designed to be scored by a veterinary nurse or technician. The chart evaluates body regions that are involved in the stress response. A total of 36 points is possible. Dogs with high scores show signs of stress and may be considered distressed, while dogs who score low may be mildly stressed. Dogs who score zero are considered calm and relaxed [13].

The same three trained raters were also responsible for scoring behavior using the CDSS via video footage of dogs during the physical examination (Consult). The raters were randomly assigned videos and instructed to rate each dog between 0 and 4 on a stress level score, across a range of dog body regions, according to the associated definitions. 

To assess inter-rater reliability for the behavioral ratings, 15 videos were randomly selected to be scored in common by all raters. This resulted in nine videos watched in common for the CDSS (physical examination only) and fifteen videos for the QBA (kennel and physical examination). To assess intra-rater reliability, each rater re-scored a random selection of 10 videos from those they had previously scored, which resulted in repeat scores on ten videos for the QBA (kennel and physical examination) and five videos for the CDSS (physical examination only).

### 2.5. Statistical Analysis

Cortisol, IgA, and IRT (ear, eye, and nose temperature) measures were fitted to a linear mixed effects model, with the parameter as the response variable, treatment, timepoint, and their interaction as the fixed effects, and the individual dog as the random effect. Since up to three replicates were run of cortisol and IgA at each sample point, these values were averaged for analysis. This was to limit any bias that might have been introduced as more samples had insufficient saliva for multiple replicates in the later timepoints. Due to the practical limitations of collecting salivary cortisol, many data were missing. The data that were collected were categorized within exposures as well as within each timepoint, and a chi-squared test was used to see if there were any significant differences within these groups for the amount of missing data. Only complete data were included in the linear mixed effects models.

TPR measures were all measured once during the consultation, and so, a linear mixed effects model was fitted with the parameter as the response variable, treatment as the explanatory variable, and dog as the random effect. Due to large amounts of missing heart rate data from the Petpace^TM^ monitors, these measures were unable to be analyzed.

Dogs were scored using the QBA at two timepoints: while dogs were kenneled prior to the consultation (Kennel) and during the veterinary health exam (Consult). These data were composed of scores ranging from 0–125 for a series of 21 different terms. These scores were summarized using a Principal Component Analysis (PCA) to create component scores (Table S1). The first three component scores were then analyzed using linear mixed effect models, with the component score as the response variable, treatment, location, and the interaction between treatment and location as the fixed effects, and dog and observer as random effects. Additionally, questions related to the dogs’ behavior were measured at a single timepoint during the vet consultation on a scale from 0 to 4 (Clinic Dog Stress Scale). These data were analyzed using Kruskal–Wallis rank sum tests to determine if there were any differences in scores based on treatment. Scores were also categorized to represent dogs that were not stressed (score 0) or stressed (score 1+) for each of the questions. These data were then analyzed using logistic mixed effects models, with the parameter as the response variable, treatment as the fixed effect, and dog and observer as random effects.

To assess the intra- and inter-rater reliability of the behavioral measures, Intraclass Correlation Coefficients (ICCs) using a two-way mixed effects model and consistency agreement were calculated for each behavior (Clinic Dog Stress Scale) and component score (QBA).

Finally, to assess the relationship between all the collected measures, a PCA was conducted. Measures taken at different timepoints were treated as separate variables for the purpose of analysis. Where multiple measures were available at each timepoint (e.g., replicates of cortisol and IgA measures, or ratings from multiple raters for behavioral measures), the mean of the values was used (Table S2). Missing values were imputed by the mean of the variable. All measures were scaled to unit variance. The first three component scores were then analyzed using linear mixed effect models, with the component score as the response variable, treatment as the fixed effect, and the dog as the random effect.

For all models, residuals were visually inspected to assess model fit. If assumptions for normality or homoskedasticity were violated, the model was re-run with a log transformation, and back-transformed means and confidence intervals are reported. In addition, where appropriate, the Tukey method was used to adjust the p-value to account for multiple comparisons.

All analyses were performed using R version 4.0.4 [42]. 

## 3. Results

### 3.1. Physiological and Behavioral Measures

#### 3.1.1. Eye, Nose, and Ear Temperature

**Figure 3:**
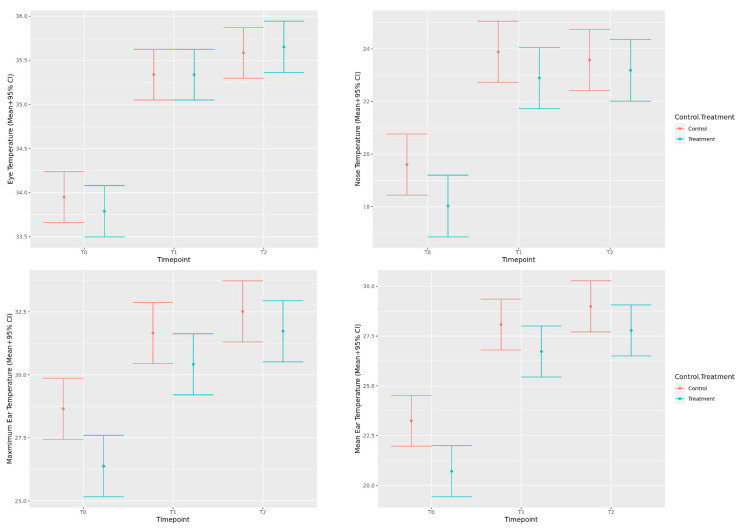
Predicted mean eye (**top left**), mean nose (**top right**), maximum ear (**bottom left**), and mean ear (**bottom right**) temperature values (+95% CI) during a mock veterinary visit measured at baseline (T0), post-kenneling (T1), and post-consult (T2) for both control visits and visits where music treatment was provided.

The models of eye, nose, and ear temperature using IRT all met model assumptions, and therefore, no transformation was required. Timepoint was significant for all the models (*p* < 0.001), with eye, nose, and ear temperatures all increasing significantly from baseline (T0) to the post-kennel (T1) and post-physical examination (T2) timepoints. However, there were no significant differences in eye, nose, and ear temperatures between the post-kennel and post-physical examination timepoints, or between the treatment and control groups at the post-kennel or post-consult timepoints (Figure 3). Dogs had significantly higher mean nose (*p* = 0.010), maximum ear (*p* = 0.004), and mean ear (*p* = 0.002) temperatures at baseline, upon entering the veterinary suite during control visits, when compared to treatment visits. These differences decreased to a tendency for mean nose (*p* = 0.098), maximum ear (*p* = 0.110), and mean ear (*p* = 0.087) temperatures at the post-kennel timepoint, and were non-significant by the post-physical examination timepoint (mean nose: *p* = 0.515; max ear: *p* = 0.311; mean ear: *p* = 0.127).

#### 3.1.2. Salivary Cortisol and IgA

**Figure 4:**
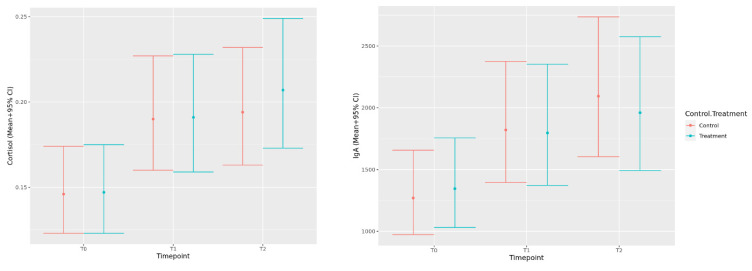
Predicted back-transformed mean cortisol (**left**) and IgA (**right**) values (+95% CI) during a mock veterinary visit measured at baseline (T0), post-kenneling (T1), and post-consult (T2) for both control visits and visits where music treatment was provided.

Chi-square analysis of the frequency of missing data for salivary measures indicated no significant differences between treatment and control groups (*p* = 0.224). However, there was a significant effect of timepoint, with fewer successful saliva collections occurring as time progressed (*p* = 0.020). Since successful collection was not associated with treatment group, we proceeded with only complete data for this analysis. When salivary cortisol and IgA data were analyzed, the residuals demonstrated a skewed distribution and heteroskedasticity, so a log transformation was used and resulted in improved model fit. The results indicated that both cortisol and IgA measurements increased significantly from baseline (T0) to the post-kennel (T1) and post-physical examination (T2) timepoints. There were no significant differences in cortisol or IgA between the post-kennel and post-consult timepoints, or between the treatment and control visits (Figure 4).

#### 3.1.3. Temperature, Pulse and Respiration (TPR)

**Figure 5:**
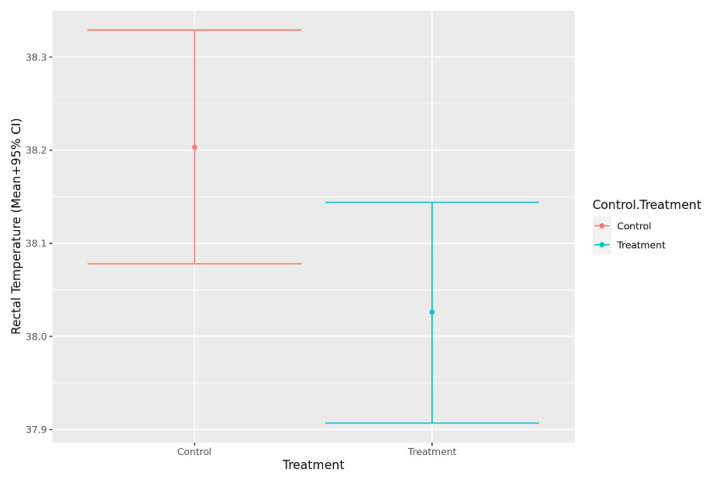
Predicted mean rectal temperature values (+95% CI) during a mock veterinary visit for both control visits and visits where music treatment was provided.

The models of rectal temperature and heart rate met model assumptions, and therefore, no transformation was required. The assumptions for normality and homoskedasticity were not met for the model of respiration rate, so a log transformation was used and resulted in improved model fit. Heart rate (*p* = 0.901) and respiration rate (*p* = 0.512) did not differ significantly between control and treatment visits. However, rectal temperature measured during the veterinary consult was significantly higher during the control visits when compared to the treatment visits when music was played (*p* = 0.009) (Figure 5).

#### 3.1.4. Dog Behavior

Analysis of the QBA data using a PCA suggested three main components of interest based on the strength of loadings and the variance explained (Table 3). The first component explained 32.2% of the total variance and was labeled “stressed/anxious”. It comprised positive loadings for “stressed”, “anxious”, “nervous” and “wary”, and negative loadings for “calm”, “comfortable”, and “relaxed”. The second component explained 20.0% of the total variance and was named “interacting/engaged”, as it comprised positive loadings for “interested”, “curious”, “excited”, “explorative”, “playful”, and “sociable”. The third component, which explained 11.3% of the total variance, was named “afraid”, and comprised positive loadings for “fearful”, “hesitant”, and “depressed”, and negative loadings for “alert”, “bored”, “reactive”, and “aggressive” did not load strongly on any of the identified components.

Inter-rater reliability between the three trained raters showed moderate agreement for scores generated for the three identified components: PC1—Stressed/Anxious (ICC = 0.69), PC2—Interacting/Engaged (ICC = 0.68), PC3—Afraid (ICC = 0.58). Intra-rater agreement demonstrated good to excellent agreement for PC1—Stressed/Anxious (ICC = 0.81–0.99), moderate to excellent agreement for PC2—Interacting/Engaged (ICC = 0.68–0.98), and poor to excellent agreement for PC3—Afraid (ICC = 0.70–0.99).

**Figure 6:**
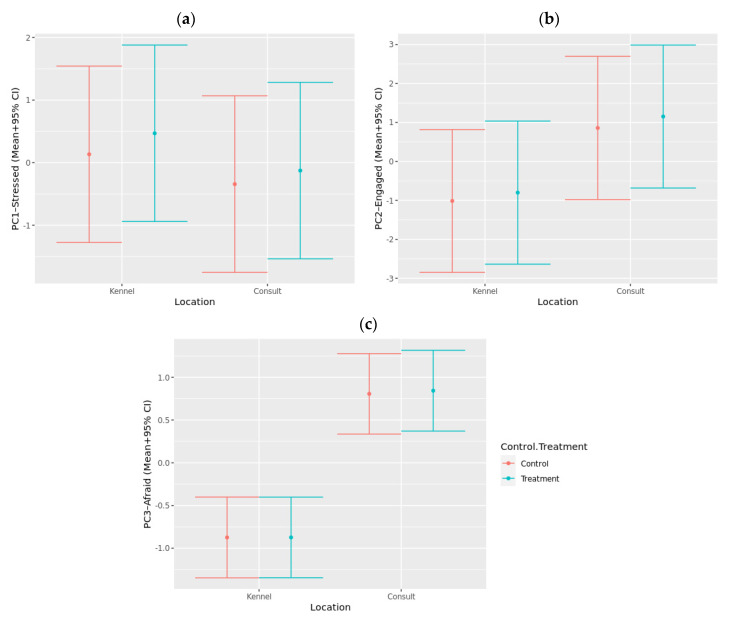
Predicted component scores (+95% CI) for (**a**) PC1—Stressed/Anxious, (**b**) PC2—Interacting/Engaged, (**c**) PC3—Afraid based on QBA ratings during a mock veterinary visit measured when kenneled (Kennel) and during a veterinary consultation (Consult) for both control visits (Control) and visits where music treatment was provided (Treatment).

When the generated component scores were analyzed using linear mixed models, they met model assumptions, and therefore, no transformation was required. There were no significant differences between the control and treatment groups for any of the components (Figure 6). Dogs scored significantly higher for the “interacting/engaged” component during the physical examination compared to when they were in the kennel (*p* < 0.001). Additionally, dogs scored significantly higher for the “afraid” component during the physical examination compared to when they were in the kennel (*p* < 0.001).

The inter-rater reliability of the behaviors measured using the CDSS ranged from poor to good depending on the behavior. Ear posture (ICC = 0.43), lips (ICC = 0.42), and gaze (ICC = 0.16) had poor agreement, body posture (ICC = 0.59) and activity (ICC = 0.54) had moderate agreement, and vocalizations (ICC = 0.83) and respirations (ICC = 0.80) had good agreement. Intra-rater reliability demonstrated that all behaviors had good to perfect agreement across the three raters (ICC = 0.83–1.00), apart from body posture (ICC = 0.71–1.00) and respirations (ICC = 0.67–1.00), which had moderate agreement to perfect agreement depending on the rater.

**Figure 7:**
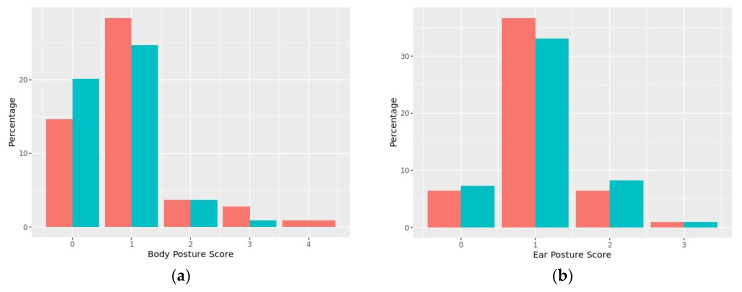

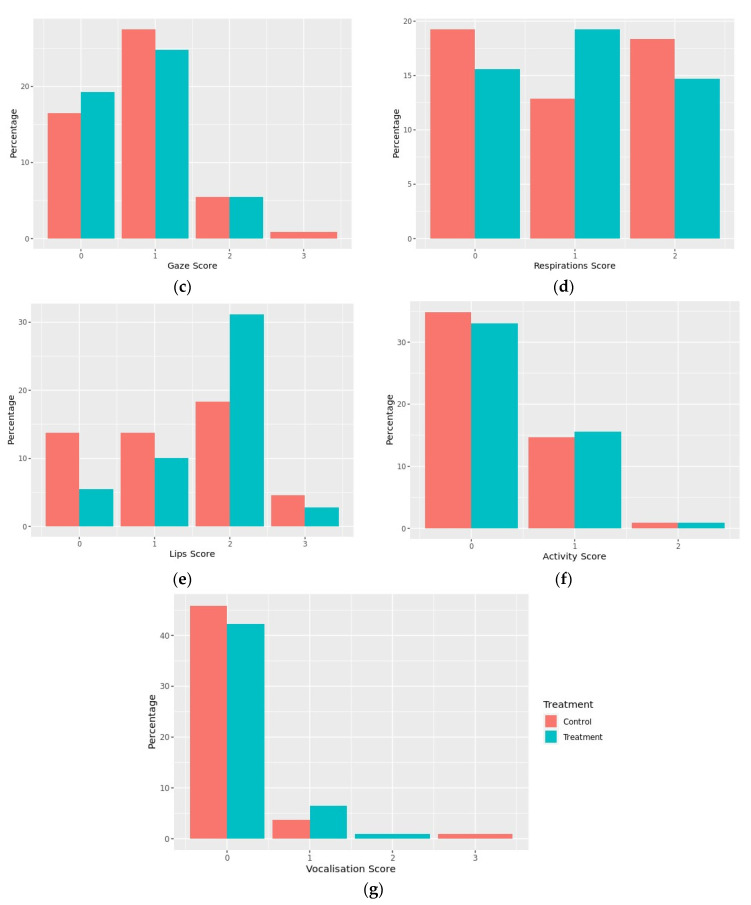
Histogram of distributions of ratings of dog behavior using the Clinic Dog Stress Scale (CDSS) during the veterinary consult for both control and treatment visits for (**a**) body posture, (**b**) ear posture, (**c**) gaze, (**d**) respirations, (**e**) lips, (**f**) activity, and (**g**) vocalizations.

No significant differences in behaviors measured using the CDSS were found between the control and treatment visits except for lips (Figure 7). There was a significant Kruskal–Wallis rank sum test for the overall lips score (*p* = 0.033), and dogs had higher odds of being scored as stressed for lips (>0) during visits with music compared to control visits (*p* = 0.020).

#### 3.1.5. Behavioral and Physiological Measures Combined

All data captured across the various timepoints throughout the mock veterinary visit were combined using a PCA. Three main components of interest emerged based on the strength of loadings and the variance explained (Table 4). The first component was labeled “temperature” and explained 20.9% of the total variance. This component comprised positive loadings for all IRT readings for mean nose, maximum ear, and mean ear temperatures, as well as rectal temperature as measured during the health examination. This component also comprised negative loadings for salivary IgA at T1 and T2.

The second component was labeled “consult stress” and explained 12.1% of the total variance. This component comprised positive loadings for the QBA “Stressed/Anxious” component during physical examination in the consultation room, the QBA “Afraid” component during consult, “activity”, “gaze”, and “body posture” from the CDSS, and salivary cortisol at T2. This component also comprised negative loadings for the QBA “Interacting/Engaged” component during the health examination.

The final component was named “kennel confidence” and explained 9.7% of the total variance. This component comprised positive loadings for IgA at T1, and negative loadings for the QBA “anxious” component during kenneling. 

**Figure 8:**
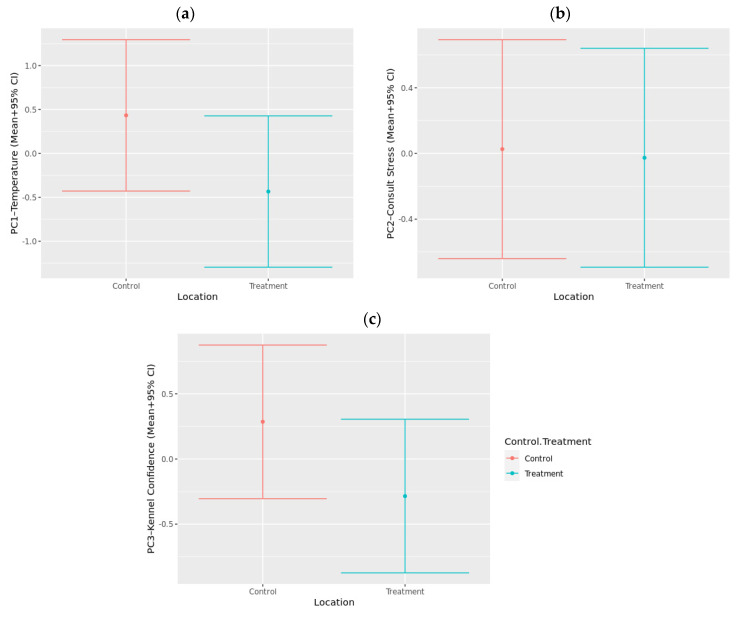
Predicted component scores (+95% CI) for (**a**) PC1—Temperature, (**b**) PC2—Consult Stress, (**c**) PC3—Kennel Confidence based all combined measures collected during a mock veterinary visit for both control visits (Control) and visits where music treatment was provided (Treatment).

When the generated component scores were analyzed using linear mixed models, there were no significant differences between control and treatment groups for any of the components (Figure 8). There was a tendency for dogs to score lower for the “temperature” component during treatment visits compared to control visits (*p* = 0.099). Additionally, there was a tendency for dogs to score lower on the “kennel confidence” component during treatment visits compared to control visits (*p* = 0.060). There was no significant effect of treatment on the “consult stress” component (*p* = 0.874).

## 4. Discussion

The aims of the present study were to understand how canine stress changes during a veterinary visit, establish the effect bespoke music has on dogs who may be experiencing stress in this environment, and highlight measures which may be of practical use to evaluate dog wellbeing within a clinical setting. Overall, the results indicated that various physiological and behavioral parameters of canine stress increased over the course of a veterinary visit. Additionally, being physically examined in a consultation room appeared to elicit more fear-related behaviors compared to when dogs were housed alone in a hospital kennel. Bespoke music designed to entrain physiological parameters did not consistently decrease measures of stress in dogs, though it was associated with a significant reduction in rectal temperature.

### 4.1. Dog Physiology during a Veterinary Visit

Several physiological measures (eye, nose, and ear temperature, salivary cortisol, and salivary sIgA) increased over time, suggesting that not only are visits to the veterinarian stressful for dogs, but they may be increasingly stressed over time. Interestingly, dogs in the control groups generally had higher baseline temperatures than the treatment groups, which is most likely a random effect due to outside temperature. This difference in temperature between groups decreased over time and was not significant at the post-kennel and post-consult timepoints. This is likely due to increased time spent indoors and the effect of stress from the veterinary examination overcoming the effect of outside temperature. To mitigate the effect of outside temperature in future studies, researchers should either measure and control for outside temperature or allow dogs to acclimate to a standardized inside temperature before taking the IRT reading. Infrared thermography has been used in other studies as a tool to evaluate canine stress in a variety of situations, from being separated from caregivers [43], to experiencing pain [44], as well as visits to the veterinarian [4,45]. However, there are inconsistencies as to whether an increase or decrease in temperature is related to negative or positive emotional valence. This may be dependent on what body part the temperature is being taken from and whether the stressor is acute or chronic. The results obtained from the present study support previous findings in which canine eye temperature increased significantly during a veterinary examination [4,45], but in contrast, another study showed ear temperature decreased [43] in response to stress caused by a separation event. To better understand whether IRT is of value as a measure of emotional wellbeing, it is necessary to further validate these measures against other stress parameters.

In the present study, both salivary cortisol and sIgA measures also significantly increased from baseline compared to post-kennel and post-consult. Again, there are challenges with how to interpret these results, as sIgA has also been shown to both increase and decrease in dogs when they have been subjected to stressful situations. For example, when exposed to loud, novel noises, sIgA concentration decreased significantly immediately after and 30 min after the noise stress [46], converse to what was observed in the current study, where sIgA concentrations increased significantly 15 min post-stressor, i.e., when left alone and being physically examined. SIgA has also been used to determine guide dog suitability, with low concentrations of sIgA being indicative of a stress response. Dogs who were considered suitable guide dog prospects had increased concentrations of sIgA over time, indicating they were better able to adapt to their surroundings and were less sensitive to stress, compared with rejected guide dogs [47]. Similarly, in a separate study, decreased levels of sIgA were observed 10 days after dogs had been introduced to novel kennel environments, and these were in the opposite direction to cortisol concentrations [48], suggesting low levels of sIgA in dogs are related to stress. However, another study demonstrated that low levels of sIgA were related to high levels of dog trainability [49], which is unlikely to be possible if individuals were experiencing stress. The measure appears to be influenced by the duration and type of stressor, as well as time of day, reflecting the complex immune response to stress. It has also been stated that the perception of the stimuli can influence results and if perceived negatively can lead to a short-term increase in sIgA concentration [50]. A recent study concluded that it is not clear whether sIgA is a useful short-term measure of stress in dogs [51]. As such, it appears more research is required to better understand this parameter. 

Similarly, interpreting salivary cortisol measures can also be challenging due to the various factors that influence this parameter [49,52], but there appears to be more consistent responses whereby cortisol concentrations increase in response to stress reflecting the activation of the hypothalamic–pituitary–adrenal (HPA) axis [12]. As such, obtaining cortisol concentrations is a common method to measure stress responses in dogs [53,54,55] and although it is affected by a range of factors, it provides valuable information on how an animal may be feeling when used in conjunction with other physiological and behavioral markers of stress [56]. The increases in salivary cortisol concentrations over time in dogs seen in the present study are similar to what has been observed in other studies [57,58,59,60] and is considered indicative of an increased stressed response, which is aligned with the direction of the IRT measures, yet, somewhat surprisingly, also the sIgA measures. In this study, there were no significant differences in cortisol concentrations at any timepoints between the two groups. Similar findings were observed when shelter dogs were exposed to classical music or no music [28]. However, in contrast to the present study, the shelter dogs spent less time standing and barking when exposed to music, suggesting music may have had a positive effect in reducing stress despite no changes in cortisol concentrations.

Core body temperature captured during the physical examination was significantly lower in the treatment group compared with the control group. This result is consistent with other studies which have previously used this measure in a range of animal species. Stress-induced hyperthermia is a common response that many mammals experience when faced with stressful stimuli [61], and core body temperature is a measure that can aid in quantifying stress responses in a range of animal species [62]. The results of this study suggest that bespoke music therapy may be successful in influencing this physiological parameter during veterinary consultations, therefore suggesting that dogs may feeling less stressed. However, it should be noted that as there were baseline differences in surface body temperature identified between the treatment groups using IRT, this difference may be spurious and not due to the music treatment. Since core temperature was only collected at a single timepoint, it cannot be determined whether there were baseline differences between the groups. A similar study found no significant differences in rectal temperature in dogs who underwent a clinical examination between groups who had owners who provided tactile and verbal interaction or groups that were provided with no interaction [4]. Despite this measure being a reliable way to evaluate stress in dogs, it may be challenging to capture as the method used (i.e., rectal thermometer) is likely to elicit mild stress in most individuals. A less invasive method would be of value. It has been demonstrated that the temperature of the tympanic membrane and ear canal measured using an infra-red thermometer is related to core body temperature [63]. Interestingly, ear temperature captured in the present study did not differ significantly between groups. However, the current study measured the temperature of the ear pinna, rather than the ear canal, which likely did not have as strong a relationship to core body temperature.

Unfortunately, due to large amounts of missing heart rate data from the Petpace^TM^ monitors, analyses of these measures were not possible. This was disappointing as these data could have provided insights as to whether the tempo of music influenced the sympathetic nervous system and entrained heart rate in dogs but also whether heart rate was related to other parameters of interest. Further research into this would be warranted, ideally with wearable devices that measures continuous heart rate and heart rate variability. Heart rate and respiration rate were also measured at a single timepoint as part of the physical examination. These measures did not differ significantly between treatment and control, suggesting there was no effect of the treatment. However, without baseline measurements, it is not known whether music affected the change in heart rate over time. Interestingly, measurements taken during the examination indicated that the mean heart rates were 103, 93, and 79 bpm for small, medium, and large dogs, respectively. These readings are all lower than the predicted heart rates used to select the music tempo and may have contributed to the music not having an anxiolytic effect. If the music was faster than the dog’s heart rate during a stress event, it is likely the process of entrainment would not have been effective in lowering the dog’s heart rate. Furthermore, the average resting heart rate estimates were selected by combining heart rate data from the relevant dog breeds used in the study at Waltham, not the specific individual dogs who participated in the study. Future studies examining music entrainment would likely benefit from capturing resting heart rate measures from the individuals being evaluated.

Despite significant changes in physiological measures over time, they are complex to interpret as various factors can influence the data which may impact how effectively canine emotional state can be inferred. As such, it is necessary to evaluate these measures in conjunction with other physiological and behavioral parameters, to better understand and assess the positive and negative valence of dogs. 

### 4.2. Dog Behavior during a Veterinary Visit

Dog behavior was captured using a QBA during the veterinary visit and a qualitative scale designed to evaluate canine stress specifically during a veterinary consultation (CDSS). There are pros and cons of using such tools, and alternative ways to measure behavior are routinely employed, such as coding of specific behaviors; however, this approach can be time consuming and is not particularly feasible to conduct in real time, or to get timely feedback. QBA is a useful method to quickly capture a holistic view of how a dog may be feeling. Recent research has suggested QBA is a reliable way of capturing behavioral data without the need for time-consuming video coding [64]. The present study relied on two qualitive scales rather than time-intensive video coding. It has been demonstrated that QBAs correlate with objective behavioral data [64], so it may not always be necessary to resort to annotating video footage to capture a range of behavioral variables. Based on the findings from this study, a number of terms were loaded together on the “stressed/anxious” component, suggesting they all measured similar responses. Inter-rater reliability for these component scores was moderate, with intra-rater reliability ranging from poor to excellent, with reliability being the highest in the “stressed/anxious” component. While observer was included as a random effect in the models in order to account for some of these differences between observers, reliability issues would still have contributed additional variation to the results and may have masked differences between the control and treatment groups. Since the QBA was being administered during a situation expected to elicit stressed/anxious responses, it is possible that reliability in the other components was low due to the infrequent occurrence of those behaviors. As such, the QBA could be refined to only include terms relevant in the components of interest. Therefore, terms from the “stressed/anxious” and “afraid” components, so seven or four terms instead of twenty, could be used in future to evaluate canine stress in a veterinary setting. However, the training of observers would be required to increase reliability.

Using output from the QBA, dogs scored significantly higher on the “afraid” component during the physical examination compared to being left alone in the kennel. Other studies support the finding that being in the consultation room can elicit fear and stress in dogs [5,8]. This is likely due to the fear-inducing nature of being restrained by a person and examined [65]. Not surprisingly, dogs scored significantly higher for “interacting/engaged” during the physical examination compared to when they were left in the kennel, suggesting that human presence and contact influences behavior. However, there were no significant differences in scores on the “stressed/anxious” component, which consisted of terms such as “stressed”, “anxious”, and “nervous”, between when dogs were left in the kennel and when they were physically examined in the consultation room. It could be expected that the veterinary examination would be more stressful for dogs; however, it is not surprising that social isolation for the population of dogs who were tested was equally stressful. These dogs are always housed with conspecifics, have regular interactions with people, and are not routinely left alone. As such, being left in a kennel on their own is likely to have induced stress. In this study, it appears the two different stressors were of equal magnitude, as measured by QBA. Interestingly, there were no significant differences between the control and treatment groups for any of the components, suggesting that bespoke music did not have a positive effect in reducing behavioral indicators of stress or fear in dogs during a veterinary visit.

Another potential alternative to time-consuming video coding is the use of simple scales that can be measured live or from video to categorize stress behaviors. The CDSS is a tool that has been developed to quantify the level of stress dogs experience during veterinary visits and examinations [8,13,66]; however, to the authors’ knowledge, this tool has not previously been assessed for reliability or validity. In the current study, the inter-rater reliability of the behaviors measured using the CDSS ranged from poor to good depending on the behavior. The behaviors that had the worst agreement were ear posture, lips, and gaze, which may have potentially been due to limitations of video angle and quality. As with QBA, observer was included as a random effect within the logistic regression models; however, reliability issues likely contributed additional variation to the results and may have masked differences between the control and treatment groups. It is possible that higher agreements could have been achieved with live scoring. As one of the objectives was to identify measures that can be easily applied into clinical practice, it would be worthwhile for future studies to further assess the reliability of the CDSS following live scoring by raters who have received intensive training. Similar to QBA, there were no significant differences in behaviors measured using the CDSS between control and treatment visits except for lips, where dogs had higher odds of being scored as stressed for lips during veterinary visits when music was played. This is opposite to what we would have expected if positive entrainment was taking place and more relaxed behavior occurred as a result. It is unclear as to why this occurred, and could be a spurious result, or may be due to multiple possible contributors to lip licking, including anticipation of food, increased salivation, or social communication. Additionally, lips had poor inter-rater agreement, which may have also contributed to the findings. There were some slight differences in body posture and gaze, indicating more dogs scored highly in the control group. This would indicate these dogs were highly stressed, suggesting music may have had a positive effect. However, these differences were small and not statistically significant so may have been due to chance. Future studies using the CDSS should consider larger sample sizes to be able to have sufficient power to detect significant differences between groups. If this tool was to be used more broadly in clinics, it is important to be aware of its limitations as a measure of stress. Future research which investigates this tool further, along with the effect music has on dog behavior during a health examination, would be of value.

### 4.3. Combining Behavioral and Phsyiological Measures

To gain a holistic view of the effect of music on the emotional state of dogs in a clinical setting, as well as how the different measures relate to each other, a PCA was run combining all the measures. The output of this PCA identified three primary components labeled “temperature”, “consult stress”, and “kennel confidence”. The first component, “temperature”, indicated that core body temperature loaded positively, along with ear and nose temperature, while IgA loaded negatively. This suggests that these parameters are likely to be measuring the same thing, related to a physiological response, possibly due to stress, activity, or external temperature. There was a tendency for dogs to score higher on this measure during control visits, indicating a potential for music to have had a stress-reducing effect. As such, this may support the use of a less invasive approach to measuring body temperature to evaluate canine stress. Surprisingly, these measures did not load on other dimensions related to canine stress or anxiety, which may suggest they are instead indicative of differences between these groups for other confounding variables, such as external temperature.

The second component, “consult stress”, indicated that measures from the CDSS positively correlated with the QBA components “afraid” and “stressed/anxious”, along with cortisol measures, and negatively associated with “interacting/engaged”, suggesting all these measures were capturing an element of canine stress during a veterinary consultation. In line with the individual results for these measures, music did not have a significant effect on this component score, suggesting that the bespoke music was not sufficient to reduce signs of stress during the veterinary consultation.

The final component, “kennel confidence”, indicated that sIgA taken after dogs had spent time in the kennel loaded positively, while the QBA “stressed/anxious” component loaded negatively, suggesting lower levels of sIgA are related to stress, as supported by other research studies [46]. When the effect of music was analyzed, there was a tendency for dogs to score lower on this component when exposed to music, indicating higher levels of stress. The findings suggest canine stress is apparent during a mock veterinary visit, and the intensity can change depending on the event occurring during the visit. It is also important to recognize that some physiological measures, e.g., body temperature, may provide more reliable data than behavioral parameters which vary considerably between individuals [67] and therefore should be included in future studies examining the effect of interventions aimed at reducing canine stress. 

Despite our hypothesis, there is limited evidence to suggest a significant positive effect of bespoke music treatment on any of the behavioral measures captured, and only one physiological measure differed significantly between the groups. These results may be due to the small sample size, the measures used, the intensity of the stressor, or that music did not have a calming effect. Similar results were obtained whereby no significant differences in behavior or physiology were observed between groups of dogs who were exposed to classical, dog relaxation music and no music within a veterinary hospital [26]. Further, it appears there is only weak evidence to suggest that classical music has the ability to reduce stress in dogs in a clinical setting [24]. Other reasons may be attributed to how the music was perceived by the dogs. This may be influenced by dog breed morphology, the shape of the dogs’ head and ears [19], as well as their hearing ability. Dogs’ hearing was not specifically tested during this study, which may have influenced the results. Additionally, surface materials, room dimensions, and room shape all influence auditory stimuli. Hard surfaces, especially metal and hard plastic, cause reflections, frequency shift with early reflections, and delays/echoes, and parallel walls induce more acoustic bounce back, which alters audio [66]. Indeed, recordings of the music being played in the kennel showed evidence of short room reverb and delays (echo effect), which could have impacted entrainment. Other clinics are likely to have similar environments and therefore would be likely to face similar limitations with acoustics. 

### 4.4. The Future of Canine Stress Evaluation and Management in a Clinicial Setting

It would be of benefit to have pets wearing non-invasive devices that rely on technology to accurately measure a range of physiological and behavioral parameters. Unfortunately, in the present study, Petpace™ was unable to provide adequate readings for analyses. Technology is increasingly being used to monitor animal welfare; this is particularly evident within the livestock industry, where a range of monitoring systems are available to farmers as a way to evaluate their animals, ensuring optimal welfare and productivity [68]. Small devices such as accelerometers are also used in the pet industry. They commonly attach to collars and accurately capture location and movement and are being developed further using machine learning to accurately detect other behaviors [69]. Recent advances in pet technology mean that these types of devices, along with other types of wearables (harnesses, etc.), are likely to be readily available in future and capable of efficiently capturing a range of data from pets. Combined with owner and/or staff questionnaires, along with the use of machine learning to automatically quantify key behaviors via video, these approaches have the potential to provide accurate evaluations of pet emotional state in real time within the veterinary environment. As such, interventions aimed at alleviating canine fear and stress can be evaluated accordingly. 

When considering music therapy as an intervention to improve pet wellbeing, it is important to be mindful of individual preferences. Some dogs may not like the music or have negative associations due to previous experiences, in which case, this could have a negative impact. One study demonstrated that auditory stimulation, in the form of classical music, improved HRV by a reduced RR variability, suggesting the novel music exposure may have had an excitatory rather than a calming effect [70]. Therefore, a more appropriate approach may be to first expose dogs to a particular type of music in a familiar and relaxing environment before they are subjected to a stressful event. For example, using the principal of classical conditioning, a neutral stimulus—music—could be played immediately prior and during activities that bring the individual pleasure and relaxation, e.g., feeding, playing, resting, and petting/attention. It could be expected that upon repeated exposures that music becomes a conditioned stimulus and produces feelings of pleasure, relaxation, and calmness. This approach has practical applications for pet owners who may wish to help calm their pet during a veterinary visit. This approach has been used with success in parents and children during a hospital visit [71] and also as a way to facilitate feeding in premature infants [72], but to our knowledge, has not been explored in detail in non-human animals. It is known that dogs’ experiences at the veterinary clinic will influence whether or not they are afraid or aggressive [73], so providing them with positive experiences will help to make them feel more relaxed. Enabling the choice to not listen to music would be of value in future studies aimed at ascertaining if music therapy (or not) improves dog welfare [19] and should be considered by professionals who currently use music in a clinical setting. 

## 5. Conclusions

The present findings suggest being left alone in a kenneled area within a veterinary clinic and subsequently undergoing a heath examination appears to be stressful for a population of dogs and these events may elicit different levels of stress. It is important to understand how clinicians, staff, and caregivers can alleviate stress in dogs during veterinary visits. It is still unclear if bespoke music has a positive effect on dogs within a clinical setting, but when designing such studies, it is important to consider study design, ensuring adequate subjects are tested and appropriate measures are captured, along with how the testing environment may impact how music is perceived by the animals. Feasible measures that can provide the accurate, real-time assessment of canine stress will help clinicians better understand the effect interventions, such as music, have on improving canine emotional wellbeing in a veterinary environment.

## Figures and Tables

**Table 1:** Qualitative behavioral analysis used to measure dog behavior in the kennel area.

Term	Definition
Aggressive	Impetuous, shows signs and posture of defensive or offensive aggression
Alert	Vigilant, inquisitive, on guard
Anxious	Worried, unable to settle or cope with its environment, apprehensive
Attention Seeking	Interactive, looking for contact/interaction, vying for people’s attention, affectionate
Bored	Disinterested, passive, showing sub-optimal arousal levels/drowsiness signs
Comfortable	Without worries, settled in its environment, peaceful with other dogs, people, and external stimuli
Curious	Actively interested in people or things, explorative, inquiring, in a positive, relaxed manner
Depressed	Dull, sad demeanor, disengaged from and unresponsive to the environment, quiet, apathetic
Excited	Positively agitated in response to external stimuli, euphoric, exuberant, thrilled
Explorative	Confident in exploring the environment or net stimuli, investigative
Fearful	Timid, scared, timorous, does not approach people or moves away, shows postures typical of fear
Hesitant	Unsure, doubtful, shows conflicting behavior, uncertain whether to approach or trust a stimulus, other dog, or person
Interested	Attentive, attracted to stimuli and attempting to approach them
Nervous	Uneasy, agitated, shows fast arousal, unsettled, restless, hyperactive
Playful	Cheerful, high spirits, fun, showing play-related behavior, inviting others to play
Reactive	Responsive to external stimuli
Relaxed	Easy going, calm, or acting in a calm way, does not show tension
Sociable	Confident, friendly toward humans and other dogs, appreciates human attentions, shows greeting behavior
Stressed	Tense, shows signs of distress
Wary	Cautious, prudent, suspicious, circumspect
Calm	Tranquil and quiet. Not showing any signs of aggression, chaotic behaviors, worry or excitement

**Table 2:** Clinic Dog Stress Scale (CDSS) used to measure dog behavior during the health examination.

Stress Level	Body Posture	Ear Posture	Gaze	Respirations	Lips	Activity	Vocalization
0	Relaxed and moves on own	High and softly forward	Will look steadily at vet	Normal—jaw relaxed	Relaxed	Flexible	None
1	Tense—can manipulate	Moving back a bit	Looks only intermittently at vet	Normal—jaw tensed	Firm	Inactive	Whine, cry
2	Rigid–hard to manipulate and a bit lower	Fully back	Will not look at vet but scans room	Panting—dry	Licking lips	Paws flexed, may tremble	Whimper
3	Hunched—hard to see or examine belly and low posture	Ears back and down	Not scanning, looking steadily at distance or owner	Panting—dripping	Yawning and licking	Periodic trembling	Snarl, snap
4	Curled—completely withdrawn and belly maximally tucked	As low and back as is possible	Staring fixedly and steadily at immediate fore-distance	Profound panting, salivating, gasping	-	Uncontrollable trembling	Bite

**Table 3:** Components extracted by the Principal Component Analysis (PCA) of Qualitative Behavior Analysis (QBA) scores. Loadings ≥ |0.50| are in bold.

Item	PC1—Stressed/Anxious	PC2—Interacting/Engaged	PC3—Afraid
Stressed	**0.915**	0.031	−0.140
Anxious	**0.879**	0.070	−0.106
Nervous	**0.789**	0.224	−0.206
Wary	**0.670**	0.126	0.428
Comfortable	**−0.** **892**	−0.126	0.054
Relaxed	**−0** **.865**	−0.167	0.028
Calm	**−0.771**	−0.373	0.140
Bored	−0.478	−0.463	0.053
Reactive	0.316	0.464	−0.177
Curious	−0.366	**0.756**	0.057
Excited	−0.270	**0.747**	0.143
Interested	−0.421	**0.746**	0.015
Explorative	−0.211	**0.698**	−0.060
Sociable	−0.317	**0.666**	0.248
Playful	−0.282	**0.665**	0.187
Fearful	0.463	−0.033	**0.780**
Hesitant	0.483	0.139	**0.725**
Depressed	0.181	−0.408	**0.538**
Alert	0.447	0.186	**−0.617**
Aggressive	0.074	0.188	0.136
Explained Variance (%)	32.2%	20.0%	11.3%

**Table 4:** Components extracted by the PCA of all measures combined. Loadings ≥ |0.50| are in bold.

Item	PC1—Temperature	PC2—Consult Stress	PC3—Kennel Confidence
IRT Nose Temp (T1)	**0.871**	0.008	−0.089
IRT Max Ear Temp (T1)	**0.793**	−0.034	0.321
IRT Mean Ear Temp (T1)	**0.782**	−0.026	0.416
IRT Nose Temp (T2)	**0.768**	−0.094	−0.125
IRT Nose Temp (T0)	**0.749**	−0.156	−0.025
IRT Mean Ear Temp (T0)	**0.704**	−0.189	0.359
IRT Max Ear Temp (T2)	**0.653**	0.029	0.250
IRT Mean Ear Temp (T2)	**0.642**	−0.070	0.423
TPR Rectal Temp (Consult)	**0.636**	0.013	−0.196
IRT Max Ear Temp (T0)	**0.586**	−0.184	0.293
IgA (T1)	**−0.527**	−0.150	**0.549**
IgA (T2)	**−0.500**	−0.049	0.460
QBA PC3 Afraid (Consult)	−0.157	**0.760**	0.276
CDSS Activity (Consult)	−0.068	**0.732**	0.181
CDSS Body Posture (Consult)	0.056	**0.728**	0.112
CDSS Gaze (Consult)	−0.070	**0.701**	0.299
QBA PC1 Stressed/Anxious (Consult)	0.199	**0.583**	−0.032
Cortisol (T2)	0.112	**0.533**	−0.397
QBA PC2 Interacting/Engaged (Consult)	0.078	**−0.618**	−0.440
QBA PC1 Stressed/Anxious (Kennel)	0.280	0.097	**−0.538**
TPR Respiration Rate (Consult)	0.466	0.410	−0.003
IRT Eye Temp (T1)	0.447	0.046	0.490
TPR Heart Rate (Consult)	0.444	0.134	−0.319
QBA PC2 Interacting/Engaged (Kennel)	0.331	0.150	−0.402
CDSS Respirations	0.321	0.245	0.026
Cortisol (T1)	0.273	0.492	−0.366
IRT Eye Temp (T0)	0.147	−0.379	0.394
IRT Eye Temp (T1)	0.125	−0.166	0.124
CDSS Lips (Consult)	0.075	0.059	0.059
Cortisol (T0)	0.057	0.176	−0.281
CDSS Vocalization (Consult)	0.045	−0.240	−0.233
CDSS Ear Posture (Consult)	−0.087	0.125	0.081
QBA PC3 Afraid (Kennel)	−0.225	0.156	0.223
IgA (T0)	−0.401	0.017	0.354
Explained Variance (%)	20.9%	12.1%	9.7%

## Data Availability

Data are contained within the article or Supplementary Materials.

## Supplementary Materials

The following supporting information can be downloaded at: https://www.mdpi.com/article/10.3390/ani12020187/s1, Audio S1: SmallDogMusic; Audio S2: MedDogMusic; Audio S3: LargeDogMusic; Table S1: QBAData; Table S2: AllData. Additionally, music playlists can be accessed for free via HealthTunes https://www.healthtunes.org/signup (accessed on 28 November 2021) and visiting healthtunes.org/code (accessed on 28 November 2021) and entering code FH2021.
